# Walk well: a randomised controlled trial of a walking intervention for adults with intellectual disabilities: study protocol

**DOI:** 10.1186/1471-2458-13-620

**Published:** 2013-07-01

**Authors:** Fiona Mitchell, Craig Melville, Kirsten Stalker, Lynsay Matthews, Alex McConnachie, Heather Murray, Andrew Walker, Nanette Mutrie

**Affiliations:** 1 Institute of Health and Wellbeing, University of Glasgow, Glasgow, Scotland, UK; 2 Glasgow School of Social Work, University of Strathclyde, Glasgow, Scotland, UK; 3 Physical Activity for Health Research Group, School of Psychological Sciences and Health, University of Strathclyde, Glasgow, Scotland, UK; 4 Institute of Health and Wellbeing, Robertson Centre for Biostatistics, University of Glasgow, Glasgow, Scotland, UK; 5 Robertson Centre for Biostatistics, University of Glasgow, Glasgow, Scotland, UK; 6 Institute for Sport, Physical Education and Health Sciences, Moray House School of Education, University of Edinburgh, Edinburgh, Scotland, UK

**Keywords:** Intellectual disability, Physical activity, Walking intervention

## Abstract

**Background:**

Walking interventions have been shown to have a positive impact on physical activity (PA) levels, health and wellbeing for adult and older adult populations. There has been very little work carried out to explore the effectiveness of walking interventions for adults with intellectual disabilities. This paper will provide details of the Walk Well intervention, designed for adults with intellectual disabilities, and a randomised controlled trial (RCT) to test its effectiveness.

**Methods/design:**

This study will adopt a RCT design, with participants allocated to the walking intervention group or a waiting list control group. The intervention consists of three PA consultations (baseline, six weeks and 12 weeks) and an individualised 12 week walking programme.

A range of measures will be completed by participants at baseline, post intervention (three months from baseline) and at follow up (three months post intervention and six months from baseline). All outcome measures will be collected by a researcher who will be blinded to the study groups. The primary outcome will be steps walked per day, measured using accelerometers. Secondary outcome measures will include time spent in PA per day (across various intensity levels), time spent in sedentary behaviour per day, quality of life, self-efficacy and anthropometric measures to monitor weight change.

**Discussion:**

Since there are currently no published RCTs of walking interventions for adults with intellectual disabilities, this RCT will examine if a walking intervention can successfully increase PA, health and wellbeing of adults with intellectual disabilities.

**Trial registration:**

ISRCTN: ISRCTN50494254

## Background

Adults with intellectual disabilities have consistently been reported to experience significant health inequalities [1-3]. Research suggests that increased mortality rates, lower life expectancy, and higher physical [4] and mental health needs [5] exist for this population. These inequalities are further accentuated by the limited success of statutory services in meeting the requirements of equality legislation and addressing the health needs of individuals with intellectual disabilities [6].

The need to support adults with intellectual disabilities to make positive lifestyle behaviour changes, as a means to health improvement, has been recognised internationally [7]. Adults with intellectual disabilities lead more sedentary and less physically active lifestyles, than the general population [8-12]. A population-based study found that adults with intellectual disabilities walked an average of 15 minutes per week [13]. Further, adults with intellectual disabilities were significantly less active than a comparison sample of adults who did not have intellectual disabilities. In addition, a recent study indicated that physical fitness levels in older adults with intellectual disabilities are much lower than in the general older population [14].

Evidence suggests that walking is an effective and sustainable form of physical activity (PA) that can be incorporated into everyday life [15]. It is also an activity that can be carried out with very sedentary or inactive populations: therefore it can be an appropriate exercise intervention for adults with intellectual disabilities. A small body of work has focussed specifically on measuring walking behaviours of adults with intellectual disabilities [16-22]. Generally, studies have used pedometers or accelerometers to measure steps walked per day. These studies suggest that adults with intellectual disabilities walk between 6481 and 11,101 steps per day [17-20]. It has been suggested that 10 minutes of brisk walking equates to roughly 1,000 steps for the general population and 800 steps for adults with intellectual disabilities [22,23]. Therefore, participants in these studies appear to be doing significantly more walking than those who were included in the population based study. Previous research has suggested that those who are already active are more likely to participate in physical activity studies than those who are inactive [24]. Consequently, a recruitment bias is likely to account for the disparity between activity levels seen in population based research and walking studies.

Interestingly, several studies that have been carried out with individuals who do not have intellectual disabilities have shown comparable recorded daily step counts. For example, a sizable population study found that 3,774 male and female participants who did not have intellectual disabilities walked an average of 7,431 and 5,766 steps per day, respectively [25]. Similarly, a walking intervention that was carried out with a low-active non intellectual disability population indicated that individuals walked an average of 6,941 steps per day at baseline and significantly increased to 8,450 steps 12 months following the intervention [26]. Although previous research indicates that some adults with intellectual disabilities may be walking within this step range, there is limited published research which has measured the intensity or cadence of walking for this population.

The current public health guidelines suggest that adults should accumulate at least 150 minutes of moderate activity per week (in bouts of 10 minutes or more). In addition, there is also the suggestion that healthy adults should aim to walk 10,000 steps/day [27]. Recently researchers have advised that different population groups should have individualised daily step targets. Accordingly, it is suggested that individuals living with a disability and/or chronic illness aim to accumulate between 6,500-8,500 steps per day with 3,000 of these steps of moderate-to-vigorous intensity physical activity (MVPA) [28].

There have been a number of studies which have examined if health intervention programmes can increase PA levels, motivation and/or other health behaviours in adults with intellectual disabilities [29-36]. However, only one pilot study of a walking intervention involving adults with intellectual disabilities has been published [37]. This is surprising given that walking is the most prevalent form of PA among adults with intellectual disabilities [13]. One hundred participants living in an institution in South Africa were invited to participate in a walking intervention three times per week, for 12 weeks, within the grounds of the institution. Participants walked for 20 minutes for the first four weeks, 25 minutes in weeks five to eight, and 30 minutes in weeks nine to 12. The study reported that participants increased their levels of PA and fitness, and reduced their percentage of body fat [37]. These results suggest that walking interventions can contribute to health improvement in adults with intellectual disabilities. However, the uncontrolled design, the lack of follow-up to examine maintenance of behaviour change and health improvement, and the institutional setting limits the generalisability of the findings. Therefore, more research is needed to assess the effectiveness of walking interventions for adults with intellectual disabilities. It would also be valuable, and respectful, to gather their views about participating in such interventions.

This study aims to add to the limited evidence base which has examined the effectiveness of walking interventions for adults with intellectual disabilities. A randomised controlled trial (RCT) has been designed which will compare the effects of a walking intervention, “Walk Well”, with a control group of individuals who have not received the intervention. This paper will present the design and rationale of the RCT and will detail the components of the Walk Well intervention.

### Aim

The overall aim of the RCT is to examine whether a walking intervention can improve the PA levels, health and well being of adults with intellectual disabilities.

The main research questions are:

1) Does a 12-week walking intervention for adults with intellectual disabilities increase the average number of steps walked per day? 2) Does a walking intervention for adults with intellectual disabilities increase the average time spent per day in moderate-vigorous intensity activity? 3) Does a walking intervention for adults with intellectual disabilities reduce time spent on sedentary behaviour? 4) Are changes in walking behaviours, physical activity and sedentary behaviour maintained at follow-up, three months after the end of the walking intervention? 5) Does a walking intervention for adults with intellectual disabilities lead to improved wellbeing and self-efficacy for physical activity? 6) How do individuals with intellectual disabilities who have participated in a walking intervention view the experience?

## Method/design

The RCT will include an active intervention group and a waiting list control group. Since this is the first controlled study of a walking intervention for adults with intellectual disabilities, a waiting list control group is preferred to a second active intervention group to obtain the clearest examination of the size of the intervention effect.

An advisory group has been set up which will involve a researcher, independent of the study, a researcher experienced in intellectual disabilities research, a family carer of an adult with intellectual disabilities and at least one individual with intellectual disabilities supported by their carer. This has been set up to ensure guidance on aspects of the study is sought from people with intellectual disabilities. Ethical approval has been received from the Scotland A Research Ethics committee.

### Study population

Participants will be invited to join the study if they are over 18 years of age and have any level of intellectual disabilities. They will be recruited from local authority day centres, provider organisations offering support to adults with intellectual disabilities or specialist intellectual disability health or local authority services in the catchment area of National Health Service (NHS) Greater Glasgow and Clyde. Participants will be excluded if they have severe challenging behaviour or needs requiring constant one-to-one support from staff, are wheelchair users or have significant mobility problems.

### Recruitment

Researchers have identified the need for a recruitment strategy in RCTs [38,39]. A strategy has been designed to guide the recruitment process, based on the framework provided by Foster et al. (2011) which identifies four key stages in recruitment; Stage 1- Pool, Stage 2- Invited, Stage 3- Responded and Stage 4- Intervention begins. The full strategy is shown in Appendix 1. Recruitment will be carried out from January 2013 to March 2014. The researchers will visit a range of services provided for adults with intellectual disabilities to ensure a representative sample of individuals with intellectual disabilities are invited to take part in the study. If individuals are interested in the study, (based on information provided by the researchers and staff) they will be given an information pack which contains details of the study. Participants can signal interest in the study by signing and returning a tear off slip in the information pack and post it using the self-addressed envelope provided. The researchers will then contact participants and arrange a visit to discuss the study.

### Sample size

There is no data from walking intervention studies involving adults with intellectual disabilities on which to base a sample size calculation. The average step count/day of 6508 (SD ± 3296) of adults with intellectual disabilities in one study is similar to the baseline step count (6802: SD ± 3212) for participants in the “Walking for Wellbeing in the West” study (WWW) [40]. The effect size of the 12-week WWW in intervention was an approximate increase of 3,000 steps/day. To take account of the different population for this study, a target increase of 2,500 steps per day due to the intervention, and a standard deviation in the step count after the 12-week intervention of 3,500 have been used for the sample size calculation. For 80% power at the 0.05 significance level, 32 participants per group would be required. To allow for a drop-out rate of 20% over the course of the study, 40 participants in each group would be required.

However, individuals recruited may live together, attend the same day centres or community activities, and be supported by the same family or paid carers. These factors would make it difficult to randomise people living together, for example, to different arms of the study. Cluster randomisation will therefore be used, but this will affect the power of the study. There is no pilot data to inform the likely degree of clustering, but assuming a conservative intraclass correlation coefficient of 0.1, and an average of 3 participants per cluster, the study sample size would need to be increased by 20%. Therefore, taking a cautious approach, a total of 50 participants will be recruited into each arm of the study.

#### Procedure

Following informed consent, a researcher will arrange a visit to collect baseline measures and provide participants with an accelerometer to wear for seven days. Individuals will be advised to continue with their normal PA while wearing the accelerometer. Accelerometer data will be used to assess baseline activity levels.

Participants will then be randomised into the active intervention or waiting list control group. For clusters of participants, the baseline data for all participants in the cluster will be collected before randomisation. The researcher will then telephone an interactive voice response system (IVRS; hosted by the Robertson Centre for Biostatistics, University of Glasgow) to register each group of participants in the study, by giving the participants’ screening number. After registering each participant, the system will notify the principal investigator of the allocation of the cluster (intervention or waiting list control). Randomisation will be stratified by the number of participants in the cluster (1, 2–3, > 4), to avoid an excessive imbalance between study groups.

**Figure 1:**
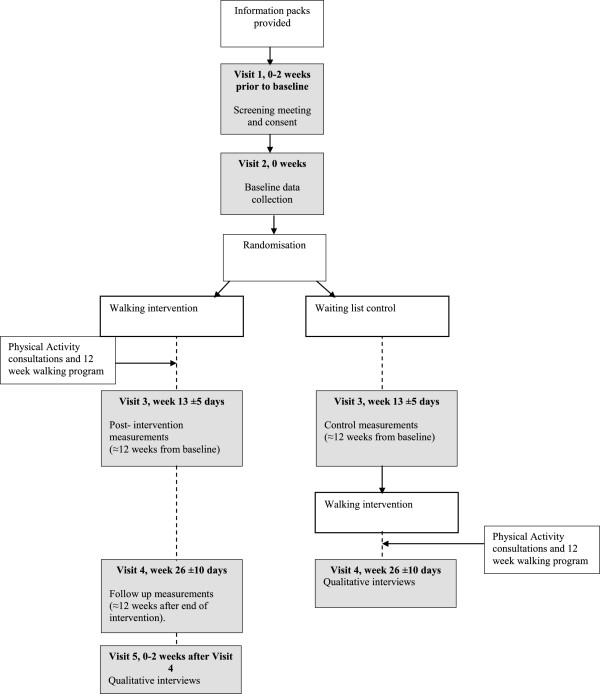
Study flow chart.

Participants who are randomised to the intervention group will be contacted by the walking advisor and arrangements will be made for the first PA consultation. Individuals who are randomised to the waiting control group will be advised to continue with normal PA until they are contacted by the researcher for 12 week measurements. The researcher collecting the data will be blind to group allocation, therefore they will not know if the participant has received the intervention. Participants will again be given an accelerometer and asked to complete various questionnaires and anthropometric measures. Participants in the control group will then be offered the opportunity to take part in the intervention. Twelve weeks later (six months from baseline) all participants will be contacted and invited to meet the researcher to complete follow up measurements. Collection of data from participants 12 weeks after the end of the walking intervention will be used to examine whether any changes in PA are maintained. Participants will have the right to withdraw from the study at any time and to decline to take part in any particular aspect or measure. This will be explained to them while seeking consent and their on-going consent will be checked and assessed throughout the intervention. The procedure is illustrated in Figure 1 below.

### The walk well intervention

The Walk Well intervention is based on the Walking for Wellbeing in the West (WWW) model used in previous studies involving adults and older adults (>65 years) who did not have intellectual disabilities [40,41]. The Walk Well intervention draws on components of the Transtheoretical Model (TTM) and other behaviour change theories (e.g. Social Cognitive Theory (SCT) [42]) and utilises current knowledge on behaviour change techniques that are associated with successful increases in walking such as self-monitoring and goal setting [43]. The intervention comprises structured PA consultations combined with a 12-week individualised walking program.

A walking advisor will be recruited to the study and trained by the researchers to carry out individualised PA consultations. The walking advisor will have previous experience in health behaviour change and will undergo a one-day training session prior to delivery of the intervention. Training will highlight behaviour change strategies guided by the TTM and other behaviour change theories including; goal setting, decisional balance, problem solving and relapse prevention. Training will be provided by members of the research team, who have extensive experience in physical activity behaviour change in adults with and without intellectual disabilities. The PA consultations will be adapted from previous consultations with adults and older adults without intellectual disabilities. These will be semi-structured and will employ a person-centred approach to ensure it is individualised to the needs of participants [44]. Participants will be invited to take part in three PA consultations during the 12 week intervention period (at the beginning, at the midway point and at the end of the intervention period).

Some adults with intellectual disabilities may require or wish a carer to go walking with them. Family or paid carers who assist participants' to walk more may also require support to change their own behaviour: therefore carers will also receive a short consultation at the same time points. Participants and carers will receive consultations at the same time, carried out in each participant’s home (or an alternative venue if preferred by participant). The length of the PA consultation will be tailored to the individual needs of each participant, and the involvement of carers in the consultation but will typically last around 45 minutes. This is in keeping with the approach to consultation sessions previously used in a weight loss study involving adults with intellectual disabilities [45].

In addition to following the MRC guidelines for evaluating complex interventions [46], the consolidated standards for reporting trials (CONSORT) [47] guidelines will be adhered to when reporting the outcomes of the RCT. Further, a simple economic evaluation to capture the costs of the intervention and compare them to the primary outcome of the trial has been designed and will be reported at the end of the study.

#### Baseline PA consultations

**Figure 2:**
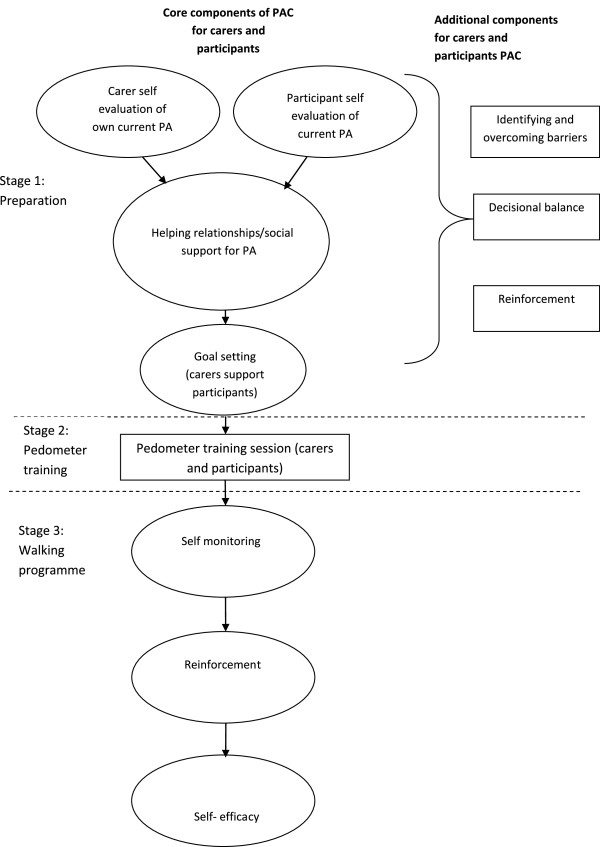
Baseline components of PA consultation for adults with IDs.

The baseline consultation framework consists of three stages; preparation, pedometer training and negotiating the initial walking programme. Within these stages, core components will be delivered to all participants and additional components will be used to tailor the consultation to individuals’ needs as required. The components in the consultation can be seen in Figure 2. Further details of these components are provided in a previous paper, describing the PA consultations [44].

The core components of Stage One (preparation) include; reviewing participants’ and carers’ current PA levels (self-evaluation of current PA). Participants and carers are then encouraged to identify what support they need for walking and who else they could ask for support, for example, other carers in the team (helping relationships/social support for PA). Carers and participants are then supported to set individualised goals for increasing walking activity together over the first six weeks of the intervention period. Firstly participants will be supported to create a week by week graded set of walking goals to work towards (goal setting). This weekly plan will aim to progressively increase the amount of time and frequency the participant is walking for. This will be tailored for each individual’s baseline PA and walking behaviours. Although it has been suggested that adults with intellectual disabilities should aim to walk between 6,500 - 8,500 steps per day with 3,000 of these steps of moderate-to-vigorous intensity physical activity (MVPA) [28], this may not be an achievable goal for those who are fairly inactive and achieve very little MVPA. Therefore, the Walk Well intervention will focus on increasing daily steps only and will encourage participants to walk at a pace which is comfortable. Individuals who are already reaching MVPA will be encouraged to continue at this level.

Participants will be provided with a pedometer and walking diary at Stage Two of the intervention (pedometer training session). The walking advisor will inform participants what a pedometer is, why they are being asked to wear it and when to wear it. The walking advisor will also demonstrate how to wear the pedometer, which functions/buttons will be used and how to record steps taken. Carers can also be provided with a pedometer to record their own walking.

As self-monitoring has been shown to support behaviour change [43,48,49], this is the first core component of stage three. Participants will be supported to monitor their steps taken in a diary provided. The behavioural methods for self-monitoring were also used in the TAKE 5 weight loss study for adults with intellectual disabilities [45] and previous walking intervention studies involving non-disabled adults. In addition, some exploratory work has been carried out by the research team which explored if and how six participants with intellectual disabilities made use of self-monitoring when provided with pedometers and diaries. Interviews with the participants and observation of the step diaries confirmed that the participants were all able to self monitor their daily steps and the pedometers increased their motivation for walking. However, it is acknowledged that this small sample of adults with mild to moderate intellectual disabilities may not be representative across a range of individuals with intellectual disabilities. Building on these findings the RCT will investigate if self-monitoring PA affects behaviour change in a range of adults with intellectual disabilities.

The walking advisor will reinforce the benefits of walking and will work to enhance participants’ self-efficacy, an important construct for PA behaviour [42]. This component of the walking programme will focus specifically on increasing participants’ self-efficacy for participating in the walking programme (including modelling and verbal encouragement). The decision to include strategies to develop self-efficacy, goal setting and self-monitoring in the core components was based on the evidence from previous behaviour change research [50-52].

Additional components of behaviour change (overcoming barriers, decisional balance and reinforcement) will be used by the walking advisor as required, tailored to the support needs of the individual. These can be used in three ways; encouraging carers to change their own behaviour, encouraging carers to support participants to change behaviour and supporting participants to change behaviour. The components that are used with each carer and participant will be recorded by the walking advisor.

#### The second PA consultation (six weeks from baseline)

The second PA consultation will review each participant’s progress towards achieving the goals set at the baseline consultation, and discuss any barriers to change that the participant and carers experience. The core and additional components described above will be used to encourage behaviour change and to reinforce knowledge about the potential benefits of PA. In addition, this consultation encourages participants and carers to maintain any increases in PA and discuss their motivation and confidence about increasing PA levels. In this consultation, participants and carers will be invited to set progressive goals for the final six weeks of the intervention period, which will be incorporated into a further six week walking programme.

#### PA consultations (12 weeks from baseline)

At the end of the 12 week intervention period the PA consultation will focus on encouraging participants to maintain any changes in walking behaviour. This will involve reviewing goals achieved and the perceived benefits of increasing walking behaviour, discussions about participants’ confidence levels (self-efficacy) about walking and a review of relapse prevention strategies that could help participants maintain increases in walking.

If a participant does not want to continue or increase their walking they will still be invited to participate in the data collection. However, if anyone expresses (verbally or non-verbally) a desire to withdraw from the study altogether, this will of course be respected. Written accessible resources on maintaining walking behaviours and PA levels will also be given to participants in the intervention group. These resources have been developed by the research team and include; a folder, a Walk Well information booklet, a step diary and a motivational DVD. There will also be a Walk Well information booklet provided for carers. Carers will be encouraged to revisit these materials regularly with participants.

### Outcome measures

#### Baseline measures

To identify any potential contraindications to the participant increasing PA, the Physical Activity Readiness Questionnaire (PARQ) [53] and demographic & health questionnaires will be completed at baseline only. If participants score positively on any item they will be advised to consult their GP about whether they should participate in the study. The following assessments will be completed by participants at the three data collection points.

#### Primary outcome - measures of PA

The primary outcome measure will be the change in average steps walked per day at 12 weeks from baseline. Secondary outcome measures will include; average number of minutes spent in PA per day (light, moderate and vigorous intensity), average time spent on sedentary behaviour per day.

To objectively measure walking behaviours and determine the time spent at various intensities of PA, all participants will be invited to wear accelerometers for seven days prior to the start of the intervention (visit two), at visit three (post intervention or control measurement) and visit four (follow up or post intervention). Actigraph GT3X accelerometers (Manufacturing Technology Inc., Florida) will be used. The accelerometer is worn at the hip, attached to a belt worn round the waist. Instructions will be given to wear the actigraph during all waking hours; except when showering, bathing or swimming. To monitor this, participants and carers will be asked to record the time when the actigraph was put on each day, any periods when it was removed, and the time it was removed prior going to bed. The actigraph has been shown to be a reliable and valid method to measure PA and sedentary behaviours [54]. Therefore, Actigraph GT3X accelerometers will measure steps taken per day, the amount of PA undertaken at light, moderate and vigorous intensity, and the amount of time spent on sedentary behaviour.

In keeping with guidelines on the validity of accelerometer data, the minimum data requirement will be set at six hours of data on at least three days from seven, to ensure a valid measure of PA levels [55]. The accelerometers will be set to record activity over 15 second intervals (epochs). Activity counts of four consecutive epochs will be summed to give activity counts for each minute. Published cut-offs will be used to express the accelerometer data as three categories of activity intensity; sedentary behaviour 0 – 499 counts per minute (cpm), light intensity activity 500 – 1951 cpm, moderate-vigorous intensity activity greater than 1952 cpm. In addition to the accelerometer data, the International PA Questionnaire-Short (IPAQ-S) will be completed by participants at all three data collection points. This will provide information about the types of activities that were undertaken. The Actigraph accelerometer and IPAQ-S have been used previously in a study involving adults with intellectual disabilities [56].

#### Secondary outcome measures

##### Well-being

Guidelines on the measurement on quality of life in adults with intellectual disabilities have been published and validated and measurement instruments developed [57].

To allow comparison with PA studies that do not include adults with intellectual disabilities as participants, the EQ–5D will be piloted as a measure of quality of life. The EQ-5D has been shown to be reliable, valid and sensitive to change in PA studies. The EQ–5D has been used as a proxy-measure of quality of life in studies involving adults with cognitive impairments due to stroke and dementia [58]. Due to the cognitive demands and the abstract nature of subjective ratings of quality of life, some adults with intellectual disabilities are unable to provide reliable subjective ratings. Since the target population of this study includes adults with the full range of intellectual disabilities, it might not be possible to collect subjective ratings of quality of life from all participants. Proxy rating and self rating of subjective quality of life have been reported to have poor agreement [59,60]. However, proxy report on objective measures on quality of life have adequate agreement with self ratings [59,60]. Hence, in this study, carers will be asked to complete the EQ–5D as a rating of the carer views of the five domains in the EQ-5D, rather than as proxy-rating. Individuals with mild intellectual disabilities will also be asked to complete the EQ-5D and the level of agreement with carer ratings examined.

To further capture any positive effects of physical activity on well being, overall vitality will be measured using the nine item Subjective Vitality Scale [61], modified for use by adults with intellectual disabilities.

##### Self efficacy

To measure changes is self-efficacy over the course of the intervention the Self-Efficacy for Activity for Persons with Intellectual Disability (SE-AID) [62] and Self-Efficacy for Exercise Scale [63] will be completed at all three time points.

##### Anthropometric measurements

Participants will be invited to have their weight, height and waist circumference measured. Measurements will be made with the participant wearing light clothes without shoes. All measurements will be made in duplicate and the final value calculated as the mean of the two measurements. Weight in kilograms (kg), will be measured to the nearest 100 grams (g), using SECA 877 scales (SE approval class III; SEA Germany). Height in metres (m) will be measured to the nearest 1 mm (mm) using the SECA Leicester stadiometer (SECA, Germany). The height (m) and weight (kg) will be used to calculate BMI using the formula; BMI = weight/height ^2^ (kg/m^2^). Waist circumference will be measured to the nearest 0.5 cm (cm) at the mid point between the iliac crest and the lowest rib, in full expiration when the participant is standing.

### Qualitative research

According to the MRC framework for the development and evaluation of complex interventions [46] qualitative research can be valuable for identifying what the important or “active ingredients” of an intervention are, and which elements are not related to the ‘treatment effect’. Therefore, semi-structured interviews will be carried out at the end of the trial. Participants with intellectual disabilities, who were able to understand and respond to questions regarding their PA participation, will be invited to take part in the qualitative interviews. Design of the set of tools has taken account of lessons learnt from previous work, taking in the views of people with intellectual disabilities, including guidelines approved by the Economic and Social Research Council (ESRC) [64].

Topics which will be covered include attitudes towards PA in general and walking in particular, including any changes in view over the 12-week intervention period, perceived benefits, drawbacks and impact of increased activity, subjective feelings of well-being before and after intervention and views about sustainability of exercise and benefit. If individuals are not able to participate in an interview, a carer or key worker will be interviewed instead. Data obtained from the latter will be treated as their views and not the proxy views of participants. With the respondent’s permission, the interviews will be recorded and transcribed. Otherwise, notes taken during interview will be written up in detail as soon as possible afterwards.

### Process measures

An in-depth process evaluation of the Walk Well intervention will be conducted following completion of the study. As recommended by the World Health Organisation [65], evaluation of health interventions is essential to inform the future implementation of effective and sustainable services. Process measures will be collected, guided by the RE-AIM framework for health interventions (Reach, Effectiveness, Adoption, Implementation and Maintenance) [66], in addition to recommendations by the World Health Organisation. Analysis of process data will provide insight into multiple aspects of the intervention, including; uptake, recruitment, promotion, effectiveness, delivery, training, resources, and sustainability.

### Data analysis

The primary outcome, change in average daily step count at the end of the intervention period (≈12 weeks) from baseline will be analysed using regression models taking account of clustering and adjusting for randomised group and baseline step count (i.e. random effects models).

Similar linear regression models will be fitted for each secondary outcome. Additional analyses may assess the effects of baseline characteristics on outcomes and investigate the evidence for interactions with treatment effects. All statistical analyses will be carried out according to a detailed Statistical Analysis Plan developed in collaboration with the Robertson Centre for Biostatistics, University of Glasgow, prior to unblinding of the randomised groups.

Analysis of interview data will involve a systematic approach set out by Holloway (1997) involving the following steps: ordering and organising the collected material, rereading the data, breaking the material into manageable sections, identifying and highlighting meaningful phrases, building, comparing and contrasting categories, looking for consistent patterns of meanings, searching for relationships and grouping categories together, recognising and describing patterns, themes and typologies, interpreting and searching for meaning. This process will be facilitated by using NVIVO, a widely used software package for ordering and coding (identifying themes) qualitative data.

## Discussion

The recent series of publications by the *Lancet* medical journal has drawn attention to the high prevalence or ‘pandemic’ of physical inactivity [67]. The series of papers highlight the harmful effects that physical inactivity and sedentary behaviour can have on the populations’ health and the environment. The need for research which focuses on increasing PA levels in specific population groups has been highlighted as an important step towards addressing this problem [68].

This paper presents the rationale and design of a RCT of a walking intervention for adults with intellectual disabilities. Since the RCT design is viewed as the optimal study design to minimise bias and provide the most accurate estimate of a complex intervention’s benefits [46], the design of this study is a key strength. As mentioned, the only other published walking intervention which has been carried out with adults with intellectual disabilities used an uncontrolled design and was carried out in an institutional setting [37]. Therefore, the Walk Well study will make an important contribution to the limited amount of work in this field. Since little is known about the feasibility of recruiting participants for walking programmes from organisations and services for people with intellectual disabilities, this study will help to identify the barriers and facilitators of using such an approach. This will be useful for future research in other PA or behaviour change programmes in this population.

Although interventions informed by the TTM have been shown to successfully support increases in PA and walking, relatively few studies have made use of the TTM to inform behaviour change interventions with adults with intellectual disabilities, many of whom have increased support needs around adaptive behaviour and experience significant social disadvantages. Furthermore, the complex health and social needs of adults with intellectual disabilities, the low baseline levels of PA and fitness [69] and the increased barriers to PA [70,71] suggests that some generic walking interventions may be inaccessible to adults with intellectual disabilities. Therefore, the design of this walking intervention for adults with intellectual disabilities is distinct from models used in previous general population studies. Consequently, exploring if components from the TTM model of behaviour change and SCT are relevant and applicable to PA in the lives of adults with intellectual disabilities is an important aspect of this study. Since these models of behaviour change recognise the importance of social support to change behaviour, they fit with the evidence-base on the importance to individuals with intellectual disabilities of support from friends, family, and paid carers, for healthy lifestyle choices and behaviours. Therefore, behaviour change theories and techniques provide a potential framework which is relevant to adults with intellectual disabilities’ lives and examining the role of family and paid carers in the process of behaviour change.

### Challenges

The anticipated challenges are based on previous work with adults with intellectual disabilities and walking studies. Since intellectual disability populations face additional barriers to PA [70,71] and have fewer choices and less control of their health than individuals who do not have intellectual disabilities [72], supporting this group to change PA behaviour may be challenging. For example, a recent weight loss study for adults with intellectual disabilities [73] found that a lack of sufficient support from carers/relatives and poor communication among carers, were identified as being barriers to change. Further, findings indicate that paid carers generally have a low level of knowledge around public health recommendations on diet and PA [74]. Interestingly, the carers did not seem to recognise the significant barriers to behaviour change for individuals with intellectual disabilities. Rather they felt that an individual’s lack of knowledge and skills and motivation were likely to prevent them changing their behaviour, rather than environmental or external barriers. Therefore carer/relative support and communication may also be a barrier to behaviour change in the proposed walking study. By anticipating this challenge, we endeavour to involve the carers/relatives in the study as much as possible.

There is some dispute in the literature about the effectiveness of identifying barriers in behaviour change interventions. For example, work carried out with non intellectual disability populations has suggested that identifying barriers to PA with participants may have a negative impact on exercise self-efficacy [50]. However, we suggest that such outcomes are likely to be related to the *process* of barrier identification, rather than this specific component of behaviour change models. Therefore, if barrier identification is applied in the PA consultations (an additional component of the intervention) a coping planning approach will be used. This focuses on identifying the ways that perceived barriers could be overcome, rather than participants merely identifying them. This psychological approach suggests that planning for how an individual might do more PA (coping planning and setting specific actions) increases the likelihood that exercise behaviour will increase [51,52].

The follow up process evaluation will provide important insight into various aspects of the implemented protocol, and will play a critical role in the development and implementation of future physical activity interventions for adults with intellectual disabilities.

Finally, as with any large scale behaviour change study, recruitment may be challenging. However, it is anticipated that our strategy for recruitment will aid this process.

## Conclusions

There are currently no published controlled studies of walking interventions for adults with intellectual disabilities. This proposed RCT explores whether a walking intervention can successfully increase daily step count for adults with intellectual disabilities. Further, if this study shows a significant increase in walking for those who undertook the PA consultations and individualised walking programme and if the individuals involved view it as a worthwhile experience, this protocol can serve as framework or model for future walking and PA interventions for adults with intellectual disabilities. This can then be tested in larger trials across different settings.

## Appendix 1 recruitment strategy framework

### Stage 1- pool

a) Identify target group within population or setting.

Adults with intellectual disabilities living in Greater Glasgow.

b) Formative evaluation of recruitment approaches

A multi point recruitment strategy will be used to recruit from three main sources:

• Adults attending local authority day centres

• Those receiving support from provider organisations

• Those using services provided by Area Intellectual Disability Teams (ALDT's) in Greater Glasgow.

### Stage 2- invited

a) Offer invitation (January 2013- December 2013)

North West (NW) Glasgow will be targeted first to assess uptake of participants local to the researcher unit.

Information packs with individual ID numbers will be given out to 4 day centres and 1 provider organisation by the researcher. These will contain a letter introducing the study and information sheets for:

a) The participant,

b) A relative and

c) A carer.

If participants would like to be contacted by the researcher with more information about the study, they are invited to sign and return the tear off slip in the self-addressed envelope provided. Staff will be asked to support individuals to read and understand the information pack, but importantly, they should not suggest how they respond. This will be explained to staff.

b) Monitor response uptake (January 2013-December 2013)

As monitoring the responses allows the researchers to evaluate the effectiveness of the recruitment strategy (Foster *et al*., 2011), the researcher will monitor how many tear slips are returned and which centre/provider the participant were recruited from (identified by the ID numbers provided). This will allow the team to asses to most effective recruitment point.

Telephone reminders have been identified as an effective strategy for recruitment (Treweek *et al*., 2010), therefore, the research secretary will phone participants who have not returned their tear off slip within a 2 week period. This active method will serve as a reminder to participants and facilitate awareness of the study. Participants can inform the secretary if they want more information about the study, or if they do not want to take part in the study. If the information pack has been lost, the secretary will send out another information pack.

Once all of the participants have either returned forms or have confirmed to the research secretary that they do not want to take part in the study, the research team will review the number of consenting participants and assess the success of the recruitment strategy.

If there are <100 participants recruited from NW Glasgow, the same strategy will be applied to another area of greater Glasgow, until the desired number of participants are recruited to the study (n ≥100).

### Stage 3- responded (February 2013-December 2013)

Participants who requested more information about the study will be contacted by the researcher to agree a date and time for a home visit. This date/time will also be agreed with a relative or nominated carer to ensure they also receive information about the study.

a) Re-invitation to responders before intervention begins

Foster *et al*. (2011) suggest that recruitment and retention to walking studies can be strengthened if participants are invited to participate face to face. Thus, the researcher will visit each interested participant in their home (or alternative venue if preferred by participant) to provide more information about the study. Consent forms and information DVD will also be given to the participants and carer/relative. Consent forms can either be filled in while the researcher is present, or these can be left with participants to allow them time to consider their participation. A carer/relative must also fill in a consent form agreeing that they will support the participant to take part in the walking programme. There will also be an opportunity for participants, carers and relatives to ask questions about the study.

b) Facilitate attendance

Evidence suggests that greater contact between trial advisors and recruiting sites may increase recruitment (Liénard 2006, Monaghan 2007). Therefore, the researcher will carry out follow up phone calls to interested participants. These will also act as reminders to participants, carers/relatives who have not returned consent forms. Participants will also be encouraged to contact the research team (or an identified colleague independent of the research team) with any other queries.

c) Establish eligibility

d) Screen participants

• Over 18 years old with intellectual disabilities living in Greater Glasgow.

• Ambulatory and able to walk unaided for 10 minutes at a time based on self/ carer report

• Any level of intellectual disabilities

• Not currently taking part in any other research study

Participants will we be screened for eligibility based on the exclusion criteria and, GP clearance (if needed, based on IPAQ scores).

e) Check all consent has been obtained

The researcher will monitor and follow up consent forms. The chief investigator will ensure informed consent is obtained before any of the specific protocol procedures are carried out.

f) Baseline measurements carried out

g) Randomisation into intervention group/ offer starting date (February 2013- Jan/Feb 2014)

Participants will be randomised into the walking intervention group or the waiting control group (randomisation by source of recruitment and level of intellectual disability).

### Stage 4- Intervention begins (February 2013- July/Aug 2014)

## Abbreviations

PA: Physical activity; RCT: Randomised controlled trial; NHS: National health service; IVRS: Interactive voice response system; TTM: Transtheoretical model of behaviour change; SCT: Social cognitive theory; MRC: Medical research council; ID: Intellectual disability; ESRC: Economic and social research council; WWW: Walking for wellbeing in the west; RE-AIM: Reach effectiveness, adoption, implementation and maintenance; BMI: Body mass index.

## Competing interests

The authors declare that they have no competing interests.

## Authors’ contributions

FM and CM led the drafting and editing of the manuscript. CM, NM, KS, LM, HM, AM and AW were involved in the original application and design of the study. All authors read and approved the final manuscript.

## Pre-publication history

The pre-publication history for this paper can be accessed here:

http://www.biomedcentral.com/1471-2458/13/620/prepub
